# Noninvasive Electromagnetic Detection of Bladder Cancer

**DOI:** 10.1155/2014/802328

**Published:** 2014-01-16

**Authors:** Luigi Cormio, Clarbruno Vedruccio, Giorgio Leucci, Paolo Massenio, Giuseppe Di Fino, Vincenzo Cavaliere, Giuseppe Carrieri

**Affiliations:** ^1^Department of Urology and Renal Transplantation, University of Foggia, Viale Luigi Pinto 1, 71121 Foggia, Italy; ^2^Italian Navy, Viale S. Bartolomeo 400, 19138 La Spezia, Italy; ^3^Department of Urology, Vito Fazzi Hospital, Piazza F. Muratore, 73100 Lecce, Italy

## Abstract

*Objectives*. Normal and neoplastic human tissues have different electromagnetic properties. This study aimed to determine the diagnostic accuracy of noninvasive electromagnetic detection of bladder cancer (BC) by the tissue-resonance interaction method (TRIM-prob). *Patients and Methods.* Consecutive patients were referred for cystoscopy because of (i) microscopic or gross hematuria and/or irritative voiding symptoms and (ii) bladder ultrasounds and urinary cytology findings negative or just suspicious of malignancy. Patients were first submitted to TRIM-prob bladder scanning by a single investigator and then to cystoscopy by another investigator blind to TRIM-prob data. *Results*. In 125 evaluated patients cystoscopy was positive for BC in 47 and negative in the remaining 78; conversely, TRIM-prob bladder scanning was positive for BC in 53 and negative in 72. In particular, TRIM-prob scanning yielded 7 false positives and only one false negative; therefore, its overall sensitivity, specificity, positive predictive value, negative predictive value, and diagnostic accuracy were 97.9%, 89.9%, 86.8%, 98.6%, and 93.6%, respectively. *Conclusions*. TRIM-prob bladder scanning was a simple and quite accurate method for non-invasive electromagnetic detection of BC. If the elevated positive and negative predictive values will be replicated in further well-designed studies, it could be used to screen asymptomatic patients at high risk of BC.

## 1. Introduction

Bladder carcinoma (BC) is the most common malignancy of the urinary tract. In the United States, more than 70,000 new cases are diagnosed every year; one out of five patients dies of this disease [1]. 

Noninvasive diagnosis of BC relies on bladder ultrasounds (US) and urinary cytology, but, whenever these investigations are negative or just suspicious of malignancy, cystoscopy remains the “gold standard” diagnostic method [2]. Cystoscopy also remains the “gold standard” followup investigation in patients with nonmuscle-invasive BC; due to the high rate of disease recurrence, these patients are likely to undergo several cystoscopies during their life [2]. Unfortunately, cystoscopy is invasive, relatively expensive, and flexible instruments have reduced but have not eliminated patients' discomfort; therefore, noninvasive diagnostic tests comparing well with cystoscopy findings would be extremely desirable.

In the last decades, the search for noninvasive tests for the diagnosis and followup of BC has concentrated onto urinary molecular tumor markers. Though some of them, such as NMP22, UroVision, and ImmunoCyt, appear particularly promising, they all remain far from the goals of having such a high positive predictive value (PPV) to avoid unnecessary workup or, most important, to have such a high negative predictive value (NPV) to avoid the risk of failing to detect tumors [2].

In this scenario, little attention has been paid to a new device for noninvasive analysis of electromagnetic anisotropy in biological tissues (the tissue-resonance interaction method, TRIM-prob; TrimProbe, Turin, Italy). Briefly, the device generates an alternating electromagnetic field that interacts with charged particles (molecules, ions, electrons, and nuclei) in a target tissue leading to a secondary radiation that varies for normal and neoplastic biological tissues [3]. Several pilot clinical studies have shown that TRIM-prob scanning may be a valuable tool in diagnosing prostate [4–8], breast [9], gastric [10], thyroid [11], rectum [12], and even bladder cancer [13].

The present study therefore aimed to determine the diagnostic accuracy of TRIM-prob in detecting BC as compared to cystoscopy, the “gold standard” diagnostic method.

## 2. Materials and Methods

Following internal institutional review board approval, consecutive patients referred for cystoscopy were informed about the study purpose and asked to provide a written consent to be enrolled. Inclusion criteria were (i) microscopic or gross hematuria and/or irritative voiding symptoms and (ii) bladder US and urinary cytology findings negative or just suspicious of malignancy. Exclusion criteria were (i) pace-makers and/or any other active implantable device, (ii) a history of previous or coexistent pelvic neoplasm, and (iii) PSA > 4 ng/mL in males.

All patients were first submitted to TRIM-prob bladder scanning by a single investigator (GL) and then to cystoscopy by another investigator blind to the TRIM-prob findings.

**Figure 1:**
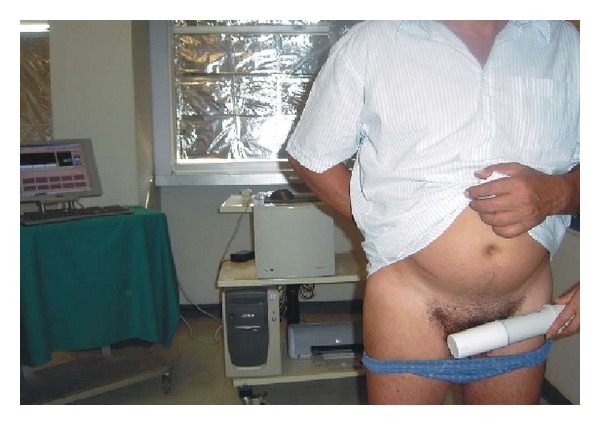
The patient stands between the probe, which is brought close to the prepubic area, and the receiver, which is located approximately 150 cm from the probe, is at the same height of the bladder.

TRIM-prob bladder scanning was carried out in a room free from relevant electromagnetic interference, including other electric device and mobile phones, and the patients were asked to remove personal metal objects. The physical principles and the technique of TRIM-prob bladder scanning have already been described in detail [13]. Briefly, the TRIM-prob system consists of a battery-operated probe, a receiver, and a computer display. The probe, which is about 30 cm long and can easily be held in one hand, contains a tuneable oscillator and an antenna emitting a very weak electromagnetic wave at several frequencies (465, 930, and 1395 MHz). With the patient in standing position and a partly full bladder, the probe is brought close to the prepubic area (Figure 1). The electromagnetic wave stimulates minute electrical oscillations that resonate inducing changes in the amplitude of one or more frequencies, depending on the pathological state of the tissue. Such changes are detected by the receiver, a multifrequency radiation pattern analyzer located at approximately 150 cm from the probe, at the same height of the bladder. Resonance values are visualized on the computer display on a logarithmic scale and expressed in arbitrary units (AU) ranging from 0 to 255. When the signal reduction at the frequency of 930 MHz confirms the exact position of the probe, the response to the 465 MHz frequency is recorded; the test is positive for BC when the signal intensity at 465 MHz is below 45 AU [13].

## 3. Results

The study population consisted of 125 patients, 100 males and 25 females, with a mean (± standard deviation) age of 62.1 ± 11.2 years. Eighty-two patients were referred because of gross hematuria, whereas 43 were referred because of irritative urinary symptoms that were associated or not with microscopic hematuria in 38 and 5 cases, respectively. Bladder US were negative in 102 patients and suspicious of bladder tumor in 23; urinary cytology was negative in 114 and suspicious of malignancy in 11.

TRIM-prob bladder scanning was negative in 72 patients and positive in 53, whereas cystoscopy was negative in 78 and positive in 47. All lesions visible at cystoscopy were small (<1 cm in diameter) and/or flat, thus explaining negative or just suspicion US findings. Comparing the findings of the two diagnostic methods, 46 (86.8%) of the 53 patients positive to TRIM-prob were also positive to cystoscopy (true positive), whereas 7 (13.2%) were negative (false positive); conversely, 71 (98.6%) of the 72 patients negative to TRIM-prob were also negative to cystoscopy (true negative), whereas only 1 (1.4%) was positive (false negative). Therefore, TRIM-prob bladder scanning had an overall sensitivity, specificity, positive predictive value, negative predictive value, and diagnostic accuracy of 97.9%, 89.9%, 86.8%, 98.6%, and 93.6%, respectively.

Of the 47 lesions visible at cystoscopy, 6 were just coagulated; of the remaining 41, 22 (53.7%) were found to be a low-grade Ta transitional cell carcinoma (TCC), 9 (22%) a low grade T1 TCC, 3 (7.3%) a Tis, 5 (12.2%) a high-grade T1 TCC, 1 (2.4%) a high-grade T2 TCC, and 1 (2.4%) a T0. 

## 4. Discussion

The dielectric characteristics of biological tissues constitute a long standing research line in the field of physics. Already in 1926, Fricke and Morse [14] evaluated the electromagnetic properties of breast cancer, benign breast tumors and normal breast tissue in human. At a frequency of 20 kHz, the frequency-dependent function called dielectric constant *ε* was tenfold greater for tumors than for normal tissue, whereas the other frequency-dependent function called conductivity *σ* was nearly the same for all tissues. The increase in *ε* for actively growing malignant tumors was attributed to an increased proportion of cell membrane surfaces per unit volume [15].

Chaudhary and coworkers [16] measured *ε* and *σ* of breast carcinoma and homologous normal tissue between 3 MHz and 3 GHz and found that both functions were always greater for tumors. However, *ε* increased towards frequencies below 30 MHz, whereas *σ* increased towards frequencies above 300 MHz. The effect on *ε* was again attributed to cell membranes, while the effect on *σ* was attributed to reorientation of dipolar particles due to the increased water content of tumors [17, 18]. It was subsequently confirmed that electromagnetic energy absorption of malignant tumors of various origins was significantly higher than that of normal tissues, particularly at frequencies around 400 MHz [19].

When the existence of significant differences between the electromagnetic properties of malignant and normal tissues was further confirmed by sophisticated *in vitro* measurements [20, 21], it became clear that a simple and noninvasive device was needed to apply these principles to cancer diagnosis. Such device for electromagnetic detection of biologic tissues anomalies, consisting of a nonlinear oscillator concealed into a cylindrical probe and a radiofrequency spectrum analyzer, was invented and patented by one of us (Clarbruno Vedruccio) who named it Bioscanner, while the commercial name TRIM-prob was imposed by the producer, Galileo Avionica a Finmeccanica company [22, 23].

Bellorofonte et al. [4] were the first to evaluate the feasibility and diagnostic accuracy of TRIM-prob scanning in detecting prostate cancer; in their study population of 757 men, the test had a 95.5% sensitivity, a 42.7% specificity, a 63.6% PPV, and an 89.8% NPV. These results have subsequently been replicated by other studies [6–8] and even by a multicenter trial [5], thus supporting also the reproducibility of the technique in detecting prostate cancer.

Similar results have been achieved in the study testing the diagnostic accuracy of TRIM-prob in detecting breast cancer [9], whereas much better results, with sensitivity and specificity up to 100%, have been achieved in the studies dealing with thyroid, gastric, and rectal cancer [10–12]. Since there is a single study for each of these organs, these findings await to be replicated by other investigators in a larger number of patients. Also for BC there is a single study in the literature comparing TRIM-prob with cystoscopy findings and showing a highly significant (*P* < 0.001) level of agreement (Cohen's *K* = 0.77) between the two diagnostic procedures [13].

The present study confirmed the high level of agreement between TRIM-prob and cystoscopy findings, as the comparison of the two diagnostic methods showed that TRIM-prob scanning had a 97.9% sensitivity, an 89.9% specificity, an 86.8% PPV, and a 98.6% NPV, with an overall diagnostic accuracy of 93.6%. The potential clinical relevance of these findings is obvious. Due to its high (86.8%) PPV, TRIM-prob bladder scanning could be used to screen patients at high risk of BC, such as smokers or people subjected to professional or environmental exposure to carcinogenic agents. On the other hand, its even higher (98.6%) NPV suggests that this test has the potential to replace cystoscopy in evaluating patients at low risk of harboring BC or in those under surveillance after transurethral resection of BC, with significant reductions in healthcare costs, patients' discomfort, and urologists, burden. Such a high NPV, ranging from 84% for prostate cancer to 100% for thyroid and gastric cancer [4–13], seems to be the common denominator in the studies dealing with electromagnetic detection of cancer and the real strength of this new technology, as it may lead to a reduction of unnecessary invasive tests or biopsies.

The present study, however, shares also some limitations of the previous similar studies. The first is the absence of “true negative” cases, as it included only patients suspected of having BC. We already designed a new study whereby patients undergoing cystoscopy as the first part of transurethral resection of prostate will serve as controls; meanwhile, it should be noticed that a previous study demonstrated a significant difference in the signal intensity at 465 MHz between patients referred for infertility and those referred for suspected prostate cancer [4]. Another limitation common to previous studies is the blind scanning of the target organ, as the system is not fused with an imaging technique. The possibility to combine TRIM-prob with US scanning of the bladder by a fusion software, thus to visualize the organ to be electromagnetically scanned, would certainly help to simplify the introduction of this new technology in clinical practice.

Specific limitations of the present study were as follows: (i) not having correlated clinical with pathological findings, but this was felt to be beyond the scopes of a study comparing two diagnostic methods and not having a centralized pathological evaluation; (ii) not having explored the problem of intra- and interobserver reproducibility of results, but this was, again, beyond the scopes of a pilot study; (iii) not having correlated the TRIM-prob diagnostic accuracy with tumor size and location, but this turned to be of little relevance in view of the extremely low rate (1.4%) of false negatives.

In spite of all these limitations, we believe that electromagnetic detection of cancer is a fascinating research field that deserves, like all new and surprising developments, scientific enthusiasm as well as critical assessment and appropriate validation. Fortunately, these steps are made easy by the fact that this new technology is, like ultrasonography, noninvasive, simple, safe (the electromagnetic radiation is 1/10 of that of mobile phones) and has low costs (approximately 50.000 Euros the machine in Italy, no disposables).

## 5. Conclusions 

TRIM-prob bladder scanning was found to be an accurate method for noninvasive electromagnetic detection of BC, having its main strength in its very high NPV. This finding was shared by all neoplasms evaluated with this new technology and, if confirmed in further well-designed studies, would suggest that the introduction of TRIM-prob bladder scanning in clinical practice could avoid a large number of unnecessary diagnostic and followup cystoscopies.
